# Cardiopulmonary function findings of pediatric patients with patent ductus arteriosus

**DOI:** 10.1097/MD.0000000000027099

**Published:** 2021-09-03

**Authors:** Hung Ya Huang, Shang Po Wang, Sheng Hui Tuan, Min Hui Li, Ko Long Lin

**Affiliations:** a Department of Physical Medicine and Rehabilitation, Kaohsiung Veterans General Hospital, No. 386, Dazhong 1st Rd., Zuoying District, Kaohsiung, Taiwan; b Institue of Medical Science and Technology, Natioanl Sun Yat-sen University, Kaohsiung, Taiwan; c Department of Neurosurgery, Kaohsiung Veterans General Hospital, No. 386, Dazhong 1st Rd., Zuoying District, Kaohsiung, Taiwan; d Department of Rehabilitation Medicine, Cishan Hospital, Ministry of Health and Welfare, No. 60, Zhongxue Rd., Cishan District, Kaohsiung, Taiwan.

**Keywords:** cardiopulmonary exercise testing, patent ductus arteriosus, physical activity, pulmonary function test

## Abstract

Transcatheter occlusion and surgical ligation are the treatments of choice for most patent ductus arteriosus (PDA) in children. Fifty-five children who had PDA completed a pulmonary function test and a symptom-limited treadmill exercise test from 2016 to 2018 at 1 medical center in southern Taiwan. The study group was divided into surgical ligation and catheterization groups, which were compared to a healthy control group matched for age, sex, and body mass index. Data about the performance on the exercise test, including metabolic equivalent at anaerobic threshold and peak, were analyzed. No differences in the pulmonary function and ventilatory parameters were observed between the surgery, catheterization, and control groups. Heart rate at peak and at anaerobic threshold significantly differed in the investigated groups. The post hoc analysis showed that the surgery group had a lower heart rate at peak and threshold compared to the catheterization and control groups (*P* = .02, *P* < .001, respectively). No significant difference was found between the catheterization group and the control group. A larger and younger group of patients were recruited, allowing for newer data about the cardiopulmonary function to be obtained. The findings suggest that patients with PDA could undergo physical training after intervention. The imposition of restrictions to limit sports activities should be avoided.

## 1 Introduction

Patent ductus arteriosus (PDA) accounts for 5% to 10% of all congenital heart disease (CHD).^[1]^ It is one of the most commonly diagnosed birth defects in pre-term infants, and it is associated with mortality and potentially harmful long-term outcomes.^[2]^ The adverse effects of an untreated PDA include: late congestive heart failure with ventricular hypertrophy; pulmonary vascular obstructive disease including Eisenmenger syndrome with shunt flow reversal, cognitive and developmental delay, infective endocarditis, aneurysmal dilatation of the ductus; and ductal calcification^[3]^ Uncertainty remains as to when or if PDA should be treated.^[4,5]^ Prophylactic and symptom-based treatments with medication, catheterization, and surgical ligation have emerged over the past decades, but these treatment strategies have yet to be proven effective.^[6]^ Surgical ligation requires exposure to general anesthesia and suspicious autonomic dysfunction.^[7]^ PDA closure via heart catheterization is theoretically less invasive. However, it has the potential to cause complications including arterial injury.^[8–10]^ There are studies to investigate the different clinical outcomes of surgical ligation and catheterization. The clinical parameters included residual leaks,^[11]^ complication rates, long-term survival,^[12]^ closure rate, and so on.

Cardiopulmonary testing (CPET) has recently gained wide acceptance as an important prognostic indicator in children with PDA.^[13]^ The crucial role of exercise testing in regard to CHD is to objectively evaluate physiological functions, and to determine the aerobic capacity of the heart. We could use CPET to assess whether complaints have a cardiac cause, and to provide physical activity recommendations. CPET provides a global assessment that better defines the cardiopulmonary state of these patients.^[14]^ In adult CHD patients, poor exercise capacity is often related to impaired heart rate response to exercise, pulmonary arterial hypertension, and the lack of pulmonary function. There is a strong correlation between adult CHD patients having poor exercise capacity and the risk of hospitalization or death.^[15]^ A cross-sectional multicenter study compared the cardiopulmonary fitness of 798 children and found that peak oxygen consumption (peak VO_2_) was lower in patients with CHD.^[16]^

To the best of our current knowledge, there is a lack of research on exercise capacity among PDA patients who received catheterization and surgery. The combination of peak VO_2_ and heart rate reserve provided the greatest predictive information after adjustments for clinical parameters such as negative chronotropic agents, age, and presence of cyanosis.^[12]^ So we hypothesized that, after catheterization and surgery, PDA patients could undergo normal physical training.

The objective of this study was to provide more evidence of the cardiopulmonary outcomes of these interventions to support the hypothesis that patients with PDA could undergo normal physical training after catheterization and surgery and to examine the influence of PDA on exercise capacity in children, to present the results of observations on the exercise test of PDA after surgical closure or catheterization.

## 2 Materials and methods

### 2.1 Patient characteristics

The study is a retrospective cohort study analyzing the data collected at 1 medical center in southern Taiwan from January 2016 to October 2018. Children aged 6 to 12 years who were referred to the pediatric cardiology outpatient clinic for follow-up of PDA were recruited. The inclusion criteria were subjects who underwent surgical ligation or catheterization, a standard 12-lead electrocardiogram, and a symptom-limited treadmill exercise test. Age, sex, and body mass index-matched children who were referred to the pediatric cardiology outpatient clinic of the Kaohsiung Veterans General Hospital between the same period for chest pain or dyspnea on exertion, and who underwent the symptom-limited treadmill exercise test without producing abnormal findings, were recruited to the control group.

We excluded patients who had structural heart disease, moderate to severe valvular disease, significant arrhythmia, ventricular hypertrophy, and pulmonary disease. Patients with missing data were also excluded. Patient demographic variables including gender, age, body weight, height, body fat, and birth echocardiography data were recorded. This study was approved by the Institutional Review Board of the Kaohsiung Veterans General Hospital (VGHKS 15-CT7-05).

### 2.2 Exercise test

Before conducting treadmill exercise testing, we explained the purpose and protocol to the subjects and their families. Informed verbal consent and written consent were obtained from the subjects and their families, respectively. We also familiarized the patients and families with the testing equipment. Then, the patients underwent the symptom-limited treadmill exercise test, in which a treadmill, a flow module, a gas analyzer, and an electrocardiographic monitor (Metamax 3B, Cortex Biophysik GmbH Co., Germany) were used, with the purpose of measuring their exercise capacity. Both groups underwent the exercise test according to the Ramp Bruce protocol that is suggested by the American College of Sports Medicine.^[17,18]^ Criteria for terminating the test were the occurrence of significant rhythm or repolarization dysfunction; inadequate increases in pulse, exhaustion, pallor, dyspnea, dizziness, and headaches; when they could no longer continue; or when they attained maximal effort.^[19]^ The VO_2_ and carbon dioxide production were measured by the breath-by-breath method during the testing. In addition, metabolic equivalent (MET), minute ventilation (VE), blood pressure, and heart rate were measured throughout the exercise test. The anaerobic threshold was determined by the VE/VO_2_ and VE/carbon dioxide production methods.^[20]^

### 2.3 Pulmonary function test

All groups underwent pulmonary function tests at rest. We evaluated forced vital capacity (FVC), forced expiratory volume in 1 second (FEV1), and maximal voluntary ventilation (MVV). Measured FVC was divided by the predicted FVC, measured FEV1 by the predicted FEV1, and measured MVV by the predicted MVV. The predicted value of each spirometry measure was calculated on the basis of spirometric reference equations for healthy children in Taiwan.

### 2.4 Statistical analysis

All statistical analyses were performed with SPSS for Windows version 19.0 (Released 2010; IBM Corp, Armonk, NY). Continuous data were presented as means and standard deviations. Categorical variables were presented as absolute numbers or percentages. Baseline characteristics of patients in the 3 groups were compared by analysis of variance with post hoc The Fisher Least Significant Difference method for continuous variables. A pre-specified 2-sided alpha of 0.05 and 95% confidence intervals were used to determine statistical significance.

## 3 Results

### 3.1 Demographic characteristics

**Figure 1:**
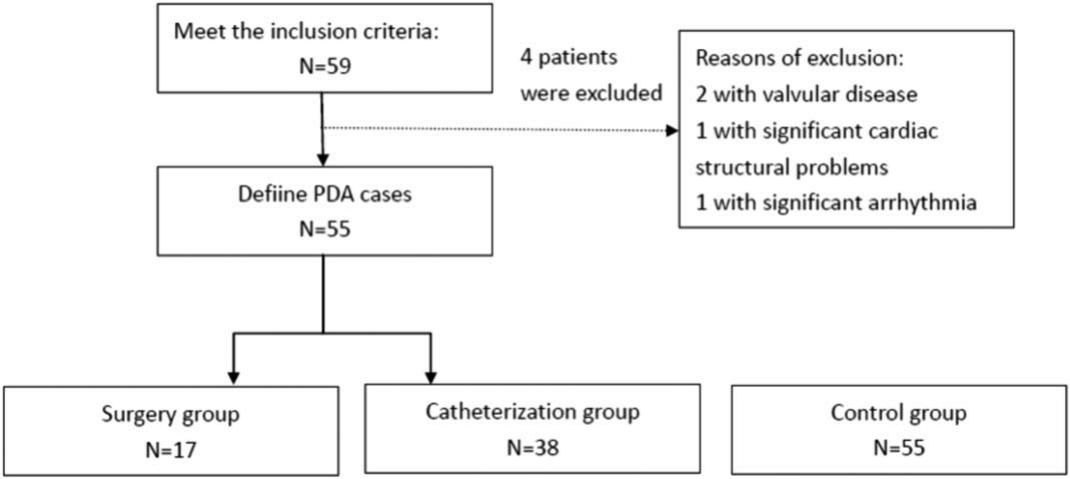
Demographic characteristics of surgery, catheterization, and control groups. PDA = patent ductus arteriorsus.

Fifty-nine patients met the inclusion criteria. Among them, 2 patients had valvular disease, 1 patient had significant cardiac structural problems, and 1 patient had significant arrhythmia. Finally, as shown in Figure 1, data of 55 patients were collected for the final analysis. Seventeen children who had surgery and 38 children who underwent catheterization were included. Table 1 shows the demographic characteristics of the surgery group, catheterization group, and control group in this study. No statistically meaningful or statistically significant differences were observed between the groups. Table 2 shows the performance of the pulmonary function test. There is no difference in the percentage of predicted FVC, percentage of predicted FEV1, and percentage of predicted MVV between the 3 groups.

**Table 1:** Demographic characteristics of surgery, catheterization, and control groups.

Demographic characteristics	Intervention type	Result	*P* value
Gender, male: female	Surgery	8:9	N/A
	Catheterization	17:21	
	Control	25:30	
Age, years	Surgery	10.12 ± 1.93	.441
	Catheterization	9.39 ± 1.94	
	Control	9.58 ± 1.93	
Height, cm	Surgery	138.10 ± 15.60	.949
	Catheterization	137.65 ± 14.33	
	Control	136.96 ± 13.31	
Weight	Surgery	35.39 ± 13.41	.732
	Catheterization	33.17 ± 10.22	
	Control	33.32 ± 9.09	
BMI, kg/m^2^	Surgery	17.76 ± 4.79	.851
	Catheterization	17.26 ± 2.67	
	Control	17.47 ± 2.48	
Body fat, %	Surgery	16.37 ± 6.91	.689
	Catheterization	17.26 ± 2.67	
	Control	17.41 ± 2.48	
Resting SBP, mm Hg	Surgery	103.65 ± 15.92	.157
	Catheterization	103.34 ± 12.96	
	Control	108.60 ± 13.99	
Resting DBP, mm Hg	Surgery	63.53 ± 8.03	.564
	Catheterization	63.13 ± 11.59	
	Control	65.35 ± 9.94	
Resting HR, bpm	Surgery	87.29 ± 12.10	.194
	Catheterization	93.21 ± 14.26	
	Control	91.45 ± 7.88	

Data are the mean ± standard deviation. BMI = body mass index, DBP = diastolic blood pressure, HR = heart rate, SBP = systolic blood pressure.

**Table 2:** Performance of pulmonary function test of surgery, catheterization, and control groups.

FVC	Surgery	1.77 ± 0.72	0.797
	Catheterization	1.82 ± 0.63	
	Control	1.88 ± 0.55	
FVCP	Surgery	88.34 ± 33.26	0.692
	Catheterization	94.15 ± 22.03	
	Control	93.57 ± 16.30	
FEV1	Surgery	2.75 ± 3.32	1.0
	Catheterization	2.77 ± 3.11	
	Control	2.77 ± 3.07	
FEV1P	Surgery	85.31 ± 29.66	0.456
	Catheterization	93.71 ± 30.79	
	Control	86.84 ± 25.06	
FVC/FEV1	Surgery	85.85 ± 12.28	0.994
	Catheterization	86.26 ± 15.98	
	Control	86.14 ± 7.98	
MVV	Surgery	38.29 ± 15.82	0.743
	Catheterization	41.68 ± 12.78	
	Control	40.31 ± 14.45	

Data are the mean ± standard deviation. FEV1 = forced expiratory volume in 1 second, FEV1P = percentage of predicted forced expiratory volume in 1 second, FVC = forced vital capacity, FVCP = percentage of predicted forced vital capacity, MVV = maximal voluntary ventilation.

### 3.2 Performance of exercise test

Table 3 showed the response of the surgery group, catheterization group, and control group to the exercise test. No statistically significant differences were observed in the routine parameters that were measured during the standard exercise test, including MET of task at the anaerobic threshold, peak MET, peak VE, peak respiratory exchange ratio, peak systolic blood pressure/diastolic blood pressure, and heart rate recovery at 1 minute after termination of the test, with the exceptions being peak heart rate (PHR, *P* < .001) and heart rate at anaerobic threshold (ATHR, *P* = .001), for which significant differences were identified between the investigated groups. In the post hoc analysis, the surgery group had lower ATHR and PHR compared to the catheterization group (*P* = .02) and the control group (*P* < .001). No significant difference was found between catheterization and control.

**Table 3:** Performance of exercise test of surgery, catheterization, and control groups.

			F value	*P* value	
Peak MET	Surgery	9.20 ± 1.70	2.518	.085	
	Catheterization	9.99 ± 1.91		.162^†^	.330^‡^
	Control	10.39 ± 2.01		.028^§^	
AT MET	Surgery	6.81 ± 1.43	2.182	.118	
	Catheterization	6.92 ± 1.56			
	Control	7.47 ± 1.37			
AT HR	Surgery	137.06 ± 19.29	7.607	.001^∗^	
	Catheterization	146.00 ± 12.82		.020^†^	.080^‡^
	Control	150.85 ± 10.35		<.001^§^^,^^∗^	
Peak VE	Surgery	71.26 ± 11.16	2.241	.111	
	Catheterization	79.27 ± 17.60		.126^†^	.514^‡^
	Control	81.73 ± 18.02		.037^§^^,^^∗^	
Peak HR	Surgery	167.59 ± 22.00	8.250	<.001^∗^	
	Catheterization	177.26 ± 12.34		.014^†^^,^^∗^	.073^‡^
	Control	182.31 ± 9.88		<.001^§^^,^^∗^	
Peak RER	Surgery	1.12 ± .07	2.963	.056	
	Catheterization	1.15 ± .08		.283^†^	.137^‡^
	Control	1.17 ± .09		.024^§^^,^^∗^	
Peak SBP	Surgery	160.13 ± 28.76	1.243	.293	
	Catheterization	150.67 ± 22.79			
	Control	157.96 ± 24.49			
Peak DBP	Surgery	85.63 ± 17.83	.459	.633	
	Catheterization	82.46 ± 21.55			
	Control	80.56 ± 17.50			
HRR	Surgery	22.86 ± 8.90	2.860	.064	
	Catheterization	33.04 ± 13.14		.021^†^^,^^∗^	.547^‡^
	Control	31.43 ± 9.22		.037^§^^,^^∗^	

Data are the mean ± standard deviation. AT = anaerobic threshold, AT MET = metabolic equivalent at the point of anaerobic threshold, DBP = diastolic blood pressure, HR = heart rate, HRR = heart rate recovery, Peak MET = maximal metabolic equivalent during exercise testing, RER = respiratory exchange threshold, SBP = systolic blood pressure, VE = minute ventilation.

∗ *P* value < .05.

† Post hoc analysis between surgery and catheterization group.

‡ Post hoc analysis between catheterization group and control group.

§ Post hoc analysis between surgery and control group.

## 4 Discussion

Our study shows that in pediatric patients with PDA, the patient who had underwent surgery or catheterization to correct PDA have mostly normal exercise capacity. However, we found that the patients who underwent surgical ligation had lower PHR and ATHR during exercise testing.

The study revealed no differences in maximum VO_2_ between the experimental and control groups, indicating that the exercise performance of patients in the experimental groups was not impaired. However, there was an observed decline in their maximum heart rate. The fact that there were no significant differences between the 3 groups in maximum VO_2_ (despite the observed decrease in the experimental groups’ maximum heart rate) could be attributed to the way in which MET is calculated (i.e., cardiac output = heart rate × stroke volume × arteriovenous oxygen difference). In addition to heart rate, cardiac output, and arteriovenous oxygen difference can also affect maximum VO_2_, such that overall VO_2_ can still be maintained even when one's heart rate is low.

Studies about the exercise performance and aerobic capacity in patients with PDA are limited. Until now, the statistical evidence remains lacking. In 1952, Lewes^[21]^ found that resting systolic pressure was higher and diastolic pressure was lower in PDA patients who underwent surgery than in corresponding controls. Nonetheless, at that time, the study groups did not follow standardized cardiopulmonary exercise testing protocol. For this reason, the management of PDA remains a controversial topic.

In the current study, we aimed to examine the influence of PDA on exercise capacity in children and to compare their physical fitness after surgical closure or catheterization. The combined use of demographic, and cardiac catheterization data with CPET provides a global perspective that further demonstrates the limitations of such patients’ cardiovascular and respiratory systems.

The ergometry data for 55 healthy children who underwent the same protocol were used as the control. For the anthropometric measurements, there is no significant difference between the healthy children and those who had undergone surgery or catheterization (Table 1).

Our findings are consistent with the limited data available from previous studies. A study by Arvidsson et al,^[22]^ compared healthy individuals to patients who had undergone surgery for congenital heart defects, and found that both groups had a similar level of cardiorespiratory fitness and maximal oxygen uptake. In 2018, Amedro et al^[16]^ had similar findings in a large cohort study. Our research results echoed those of the 2 studies, which is that such patients’ cardiorespiratory capacity is comparable to those of normal people. However, while those studies had examined CHD patients at various levels of severity, the present study had instead focused only on PDA patients.

Norozi et al^[23]^ evaluated the maximal exercise capacities of children with congenital heart defects and compared them to their peers. The children with simple heart defects achieved comparable maximal cardiopulmonary performance, whereas those with more complex conditions such as Tetralogy of Fallot and single ventricle function encountered greater functional limitations, depending on the severity of their heart defect. Most studies involved a broad spectrum of CHDs and a small number of PDA patients. The few available studies on this subject are contradictory.

In patients who underwent surgical repair, heart rate dynamics during exercise are often abnormal.^[24]^ Significant reductions of maximal heart rate in patients who had surgical ligation may indicate that chronotropic incompetence, and failing to reach the age adjusted maximum heart rate, may play a role in the pathophysiology in this population. Sinus node dysfunction, which is caused by cardiac denervation with impaired sympathetic reinnervation and damage to the sinus node or the vascular supply, is a condition that could explain this finding. It is defined as the inability to increase one's heart rate adequately to meet metabolic demands and may be related to alterations in sympathetic tone, as well as autonomic abnormalities.^[25]^ Studies have also shown that impaired chronotropic response during exercise treadmill testing is correlated to increased risk of major adverse events and all-cause mortality.^[26,27]^

von Scheidt et al^[28]^ detected impaired chronotropic response. In their cohort of 103 pediatric patients with CHD, PHR was lower in the CHD group than in controls. Massin et al^[29]^ found chronotropic impairment after surgical closure in patients with atrial septal defect. In that study, exercise heart rate at peak was significantly reduced in the surgery group. Heart rate dynamics during exercise are often altered in operated CHD. The author concluded that chronotropic impairment is less prominent after transcatheter closure of atrial septal disease. The results of these studies corresponded to our findings.

Exercise intolerance is common in adults with CHD and predicts hospitalization and mortality.^[30]^ It is affected both by cardiac anatomy and physiology.^[15]^ Changes in cardiopulmonary function of patients who had surgical ligation may put a limit on their capacity to exercise, which would play an important role in the variation in PHR between patients. Most experts have agreed that 3 months after PDA closure, asymptomatic patients with normal cardiac examination can participate in all forms of competitive physical activity.^[31]^ However, in the case of some patients and their parents, complications relating to congenital heart defects and attitude toward exercise can have an effect on a patient's self-confidence with respect to physical activities. Another frequently encountered problem is overprotection, which could be the reason why such patients can usually perform on par with their healthy peers for low-intensity exercises, but then find it challenging to maximize their exercise intensity.^[32]^

The cardiopulmonary test in our patient group showed similar capacity in patients with PDA and the normal peers. The result of exercise testing could increase motivation and confidence in children and parents. Thus, we suggested that patients with PDA could reach maximal exercise intensity. Therefore, there is a necessity in physical training.^[32]^ The imposition of restrictions to limit competitive sports and exercise should be avoided.^[33]^

This study has the following limitations. First, the criterion for maximum VO_2_ during exercise testing was calculated according to the American College of Sports Medicine guidelines.^[17]^ However, no existing studies have discussed the criteria for achieving O_2_ max during exercise testing in children with CHD. Therefore, it is unclear which criteria are suitable for determining whether a study group has reached O_2_ max. Second, for patients with PDA, medication was a choice of treatment. Because our patients were referred by the pediatric cardiology outpatient department, patients who received medication treatments were not recruited. In addition, data relating to birth conditions were not accessible for analysis and, therefore, not discussed in the study. Lastly, the participants were recruited from 1 medical center. A larger national-level study is needed for further evaluation as our results might not be applicable to the patient population of Taiwan as a whole.

## 5 Conclusions

A larger and younger group of patients were recruited, allowing for newer data about the cardiopulmonary function to be obtained. The findings suggest that patients with PDA could undergo physical training after intervention. The imposition of restrictions to limit sports activities should be avoided.

## Author contributions

**Conceptualization:** Hung Ya Huang.

**Data curation:** Hung Ya Huang.

**Formal analysis:** Hung Ya Huang.

**Investigation:** Shang Po Wang.

**Methodology:** Shang Po Wang.

**Project administration:** Min Hui Li.

**Software:** Shang Po Wang.

**Supervision:** Sheng Hui Tuan.

**Validation:** Sheng Hui Tuan.

**Visualization:** Sheng Hui Tuan.

**Writing – original draft:** Hung Ya Huang.

**Writing – review & editing:** Ko Long Lin.
